# Protocol for a randomised controlled trial investigating the effectiveness of an online e health application for the prevention of Generalised Anxiety Disorder

**DOI:** 10.1186/1471-244X-10-25

**Published:** 2010-03-21

**Authors:** Helen Christensen, Kathleen M Griffiths, Andrew J Mackinnon, Kanupriya Kalia, Philip J Batterham, Justin Kenardy, Claire Eagleson, Kylie Bennett

**Affiliations:** 1 Centre for Mental Health Research, School of Health & Psychological Sciences, College of Medicine, Biology and Environment, Australian National University, Australia; 2 ORYGEN Research Centre, University of Melbourne, Australia; 3 Centre for National Research on Disability and Rehabilitation Medicine, Mayne School of Medicine, University of Queensland, Australia; 4 Brain & Mind Research Institute, University of Sydney, Australia

## Abstract

**Background:**

Generalised Anxiety Disorder (GAD) is a highly prevalent psychiatric disorder. Effective prevention in young adulthood has the potential to reduce the prevalence of the disorder, to reduce disability and lower the costs of the disorder to the community. The present trial (the WebGAD trial) aims to evaluate the effectiveness of an evidence-based online prevention website for GAD.

**Methods/Design:**

The principal clinical question under investigation is the effectiveness of an online GAD intervention (E-couch) using a community-based sample. We examine whether the effect of the intervention can be maximised by either human support, in the form of telephone calls, or by automated support through emails. The primary outcome will be a reduction in symptoms on the GAD-7 in the active arms relative to the non active intervention arms.

**Discussion:**

The WebGAD trial will be the first to evaluate the use of an internet-based cognitive behavioural therapy (CBT) program contrasted with a credible control condition for the prevention of GAD and the first formal RCT evaluation of a web-based program for GAD using community recruitment. In general, internet-based CBT programs have been shown to be effective for the treatment of other anxiety disorders such as Post Traumatic Stress Disorder, Social Phobia, Panic Disorder and stress in clinical trials; however there is no evidence for the use of internet CBT in the prevention of GAD. Given the severe shortage of therapists identified in Australia and overseas, and the low rates of treatment seeking in those with a mental illness, the successful implementation of this protocol has important practical outcomes. If found to be effective, WebGAD will provide those experiencing GAD with an easily accessible, free, evidence-based prevention tool which can be promoted and disseminated immediately.

**Trial Registration:**

Controlled-trials.com: ISRCTN76298775

## Background

Generalised Anxiety Disorder (GAD) is a disabling mental illness. Approximately 5% of the general population experiences the disorder at least once in their lifetime [1], with populations surveys indicating a lifetime prevalence rate of between 4.3-5.9% and a 12 month prevalence rate of between 1.2-1.9% [2,3]. Although little data is available, best estimates suggest that the annual incidence rate for GAD is 1.8% [see [4]].

GAD is characterised by prolonged excessive worry within numerous domains, restlessness, fatigue, difficulty concentrating, irritability, muscle tension, and sleep disturbance [5]. It can be highly debilitating and has a significant burden on the community largely due to low rates of treatment sought as well as a shortage of therapists identified in Australia and around the world [6]. Data from the US National Comorbidity Survey indicates that only approximately 37% of those surveyed reported seeking treatment services for GAD [7]. Sub-threshold anxiety (i.e., elevated symptom levels which fall short of criteria for clinical diagnosis) is also common with a prevalence of 3.6% in the population. It is also associated with suicide attempts, and work impairment, and has not been found to differ substantially in profile from clinical GAD [8].

The cost of GAD to the community is elevated as a result of its chronic course [9]. GAD frequently presents early in the lifespan and affects the individual throughout adulthood, with an estimated lag time to treatment of between 9 and 23 years [10]. Consequently, effective prevention in young adulthood has the ability to reduce ongoing disability and costs to the community [11,12].

There is some evidence that GAD can be prevented either through a focus on salient risk factors such as anxiety sensitivity [13], pessimistic thinking [14], family history [15], withdrawn or inhibited temperament [16], known as *selective *prevention programs [17], or by targeting sub-threshold symptoms in those who do not meet diagnostic criteria for the disorder (*indicated *prevention programs) [18]. However, very few programs with a genuine preventive focus have been conducted with young adults, and rarely have prevention programs investigated the reduction in the number of incident cases. According to the Institute of Medicine (IOM) criteria [19], true prevention trials are those that exclude individuals meeting criteria for the disorder. In adults, there are only two such trials [14,17] and no indicated trials, although one targeting the very elderly is currently in progress [20]. Trial data from school programs combined with other prevention studies indicates that prevention rates vary, depending on the recruitment and prevention strategy, intervention type, length of follow-up or sample age. Findings from these studies indicate that the percentage of participants without intervention meeting diagnostic criteria at follow-up are higher (8-54%) than those exposed to prevention programs (1-20%). However, these trials to date provide a scant evidence base on which to build practical prevention programs for adults or to provide unequivocal evidence for the benefit of prevention programs outside of school environments. A definitive prevention trial in young adults is needed. A trial of this sort provides the opportunity to establish the benefit of prevention, and also to increase knowledge about the etiological factors that predict conversion to GAD.

The study protocol presented here provides a description of the background to the WebGAD trial including a description of: 1) the benefits of web-based interventions; 2) the effectiveness of web-based interventions for mental disorders; and 3) the nature of attrition. A description of the methods, design, and current status of the trial is also included as is a discussion of the possible implications that may arise from the findings.

### Benefits of Web-based Interventions

Web interventions have distinct advantages with respect to prevention where easily implemented, cost effective, high volume interventions are needed simultaneously by large numbers of individuals. Evidence suggests that web-based interventions are often preferentially sought for the anonymity, their lack of face-to-face contact, and their capacity to be used privately at home [21]. They may 'increase participation likelihood among individuals who might not otherwise seek care' [22]. Internet interventions - if automated - are able to deliver psychiatric intervention with fidelity, giving them an advantage over other programs. The use of the internet in health has high acceptability, with over 38% of Americans reporting that the internet has helped the way they take care of their health [23]. This trend strongly indicates that people are increasingly taking a central role in the management of their own health and evidence suggests that self-help techniques are effective in the treatment of mental disorders [24]. In addition, it has been reported that people are increasingly turning to the internet for information specifically on mental health [25].

### Effectiveness of Web-based Interventions for Mental Disorders

There is evidence that web based interventions (often in combination with therapist input) are effective for a range of mental health symptoms including depression [26,27], panic [28,29], post traumatic stress disorder (PTSD) [30], perceived stress in schizophrenia [31], stress [32], insomnia [33], and eating disorders [34,35]. As noted above, the effectiveness of these applications for the prevention of GAD has not been evaluated.

### Attrition

An important challenge for web-based interventions is the high rate of attrition. Evidence [35] suggests that attrition rates for web-based programs are quite high. A number of factors have been identified that can improve adherence to e-health programs, including push factors, or 'tracking', which include reminders to visit or return to websites, and personal contact through face-to-face or phone contact with service providers or trial researchers. Rewards or enhancements for engagement with the site or service, and endorsement and feedback by professional health care providers have also been found to increase retention rates [36]. Comparison of the outcomes of two trials of a depression website suggested that support (weekly telephone follow-up with instructions to visit the website) resulted in substantially higher module use than the same intervention without such contact [37]. Trials of another 'overcoming depression' website reported that reminders (telephone and email) were likely to be the crucial factor in determining retention (and improvement) [26]. However, overall, there has been little systematic investigation of factors which promote adherence in a range of mental health conditions. For this reason, there is a clear need to examine whether web interventions are enhanced by support. Thus, the WebGAD study will investigate the effect of telephone and email reminders *versus *no contact on retention rates.

### Prevention condition: "E-couch"

In the absence of research indicating reliable risk factors for the onset of anxiety, and given that our sample was selected on the basis of symptoms, our approach for this trial is to offer a preventative program that included components found effective for both the treatment of GAD, and in the prevention of GAD. As noted above, data from prevention trials are relatively weak, given the small number of completed trials. The intervention, E-couch, will be delivered as a 10-week multimedia internet application. The E-couch program is comprised of four sections - psycho-education, CBT, relaxation and exercise. Research indicates that all four components are effective in reducing anxiety levels [34,38].

*Psycho-education *will be covered in weeks 1 and 2. It contains information about the definition of worry; its distinction from stress and fear; the differentiation of GAD from Panic Disorder, Specific Phobia, Separation Anxiety Disorder, Adjustment Disorder, and PTSD; prevalence rates; the problem of comorbidity and information on medical, psychological, and lifestyle treatments for anxiety. The psycho-education section is modelled on mental health literacy interventions that have been shown to improve attitudes to and reduce symptoms of depression and anxiety [27]. It is based on clinical practice guidelines [39] as well as on reviews of evidence of alternative and lifestyle treatments [34]. The *Cognitive Behaviour Therapy (CBT) *toolkits will be introduced in weeks 3, 4, 5, 6, and 7. The CBT toolkits are designed to address typical anxious thoughts and targets worry-related thoughts and beliefs [40]. The CBT component for anxiety is based on previously developed materials which have established efficacy for anxiety cognitions and beliefs in at-risk individuals [13,41]. The third section of the E-couch intervention provides two *Relaxation Exercises*. These will be downloadable from the site during weeks 8 and 9 of the intervention, although they are freely available at any time. Mindful Meditation is a type of meditation which involves using awareness of breathing to keep a focus on the present moment. The Progressive Muscle Relaxation (PMR) component, aims to induce a relaxation response through systematic relaxation of the body. It involves participants progressively tensing and relaxing each muscle in their body, whilst also paying close attention to feelings of tension and relaxation. The *Physical Activity *intervention introduced in week 10 but lasting for longer than a week, uses walking, tailored to stages of change in participants' level of fitness.

### Attention Control Condition: "HealthWatch"

HealthWatch is an online program first developed for the ANU WellBeing Study [42]. In the form employed in the current study it provides information about various health topics each week for 10 weeks. These cover environmental health, nutrition myths, heart health, activity, medication, the effects of temperature, oral health, blood pressure and cholesterol, calcium, and back pain. To encourage interaction, participants are also asked to respond to a number of questions about potential risk factors for anxiety. Preliminary evidence from the WellBeing research trial suggests that the site is not associated with a reduction in depressive or anxiety symptoms over time.

Participants in the HealthWatch or E-couch conditions will complete the 10 week online program at their own leisure at home or office. Each module will last between 30 and 60 minutes and will be deployed weekly. If participants in the E-couch condition wish to continue using the program after the intervention period, they have the option of accessing it through the open-access website.

## Methods/Design

### Design of the WebGAD Trial

The study is designed as a five arm randomised controlled trial with three active interventions and two comparators. There will be five measurement occasions: screening, baseline, post-test, and follow-ups at 6 and 12 months after the post-test survey. This study was granted ethical approval by the Australian National University Human Research Ethics Committee (protocol number 2008/548). If approved by our ethics committee, an additional 2 year follow-up period will be included. Scales that will be administered at each time point are listed below in Table 1.

**Table 1:** Scales to be administered at each measurement occasion

	Screening	Baseline	Post-test	6 month	12 month
Demographics	X				
GAD-7	X	X	X	X	X
Prototypes	X			X	
Generalised Anxiety Stigma Scale	X		X	X	
Self-perceived emotional health	X			X	
Social Phobia Inventory	X		X	X	
Patient Health Questionnaire Panic	X		X	X	
Patient Health Questionnaire Depression	X		X	X	X
Kessler 10	X		X		X
Panic & Social phobia screeners	X		X	X	
Anxiety Literacy Scale	X		X	X	X
Eligibility	X				
Anxiety Sensitivity Index		X	X	X	X
Penn State Worry Questionnaire		X	X	X	X
Centre for Epidemiological Studies Depression		X	X	X	X
Days out of role		X	X	X	X
Perceived helpfulness of sources		X	X	X	X
Help seeking		X	X	X	X
Medical Outcomes Study Social Support Survey		X			
Childhood adversity		X			
Life events		X			
Alcohol Use Disorders Identification Test		X	X	X	X
Physical health		X	X	X	X
Medications		X	X	X	X
Smoking		X	X	X	X
Beliefs about internet		X	X		X
Usefulness of E-couch			X	X	X
Condition preference		X			
Contamination			X		X
Employment status items (in demographics)					X

Note: For details of the references for these measures please see below.

### Recruitment & Inclusion/Exclusion Criteria

Recruitment will take place in two steps. Step 1 will involve a screening assessment, mailed to individuals aged 18-30 years randomly selected from the Australian Electoral Roll. In Australia, it is compulsory for all Australian citizens aged 18 years or older to be registered on the Commonwealth Electoral Roll. Randomly selected individuals will be screened for symptoms using the GAD-7 [43] and individuals with GAD-7 scores 5 or greater will progress to step 2.

Step 2 will involve the administration of the MINI diagnostic interview [44] to exclude individuals with a diagnosis of current GAD (and other relevant diagnoses). Diagnoses based on the MINI will result in referral. Participants ineligible to take part in the prevention trial due to a positive GAD diagnosis will be offered the opportunity to take part in the WebGAD Treatment trial being conducted by the Brain & Mind Research Institute at the University of Sydney (ISRCTN76298775) (Christensen, Guastella, Mackinnon, Griffiths, Eagleson, Batterham, Kalia, Kenardy, Bennett, & Hickie: Protocol for a randomised controlled trial investigating the effectiveness of an online e-health application compared to attention placebo or sertraline in the treatment of generalised anxiety disorder, Submitted). A complete list of inclusion/exclusion criteria can be found in Table 2. The study will aim to recruit a total of 600 participants (120 for each of the trial arms). Recruitment will be carried out in four intake cohorts over 12-18 months.

**Table 2:** Inclusion/Exclusion Criteria for WebGAD Prevention Trial.

Inclusion Criteria	Exclusion Criteria
18-30 years old	Currently undergoing CBT or seeing a psychologist/psychiatrist
Score ≥ 5 on the GAD-7 scale	Current or previous diagnosis of Bipolar Disorder, Schizophrenia, or Psychosis
Consent to participate in the study	At risk of self-harm or suicide based on the MINI depression module
Do not meet criteria for GAD on MINI GAD module	Current diagnosis of panic disorder, social phobia or post-traumatic stress disorder according to MINI criteria
Provide an active email address and phone number	Currently on psychiatric medications
Sufficient English language literacy	
Access to the internet (home or work)	

Since depression and anxiety are substantially correlated, depression is not an exclusion criterion for the trial, nor is personality disorder. However, participants who meet criteria for Panic Disorder, Social Phobia or PTSD will be excluded and offered treatment through the clinic at the Brain & Mind Research Institute.

### Components of the Five Trial Arms

The Prevention arm of the WebGAD study consists of five experimental conditions. Three of these involve the provision of the active intervention, E-couch. The two HealthWatch control conditions serve as attention and assessment matched credible comparator/placebo interventions. E-couch will be delivered as a 10-week web intervention with minimal contact. It will be delivered either **a) **on its own, with no telephone or email reminders, or **b) **with weekly automated emails serving as reminders and containing supportive messages, or **c) **with weekly telephone calls, which prov---ide supportive messages and reminders. The control condition, HealthWatch, matched for participant involvement will be delivered either **d) **alone with no reminders or phone calls, or **e) **in conjunction with weekly telephone calls.

A comparison of outcomes under conditions **(a) **and **(d) **will establish the basic effectiveness of the intervention. The remaining conditions will test whether the effectiveness of the E-couch program can be enhanced with support, and if so, what kind of support. Conditions **(c) **and **(e) **can determine whether any prevention benefit found is attributable to the contact and support offered through telephone calls or to the intervention itself. The email condition, **(b)**, will establish the effectiveness of automated support. This has critical implementation implications because automated internet applications are cheap and easy to disseminate.

### Study Hypotheses

• It is hypothesised that E-couch online therapy, compared with the attention control condition, will reduce symptoms of anxiety, prevent the development of GAD, reduce worry, and depression, improve mental health literacy, enhance help seeking and improve other secondary outcomes.

• The addition of support for participants undergoing E-couch therapy, either in the form of automated emails or telephone calls, is expected to have a greater impact on participants' anxiety levels than E-couch alone.

• E-couch therapy plus weekly telephone support will have greater effect than weekly telephone support in the context of the control condition.

• In terms of support, E-couch plus weekly telephone support will not be significantly inferior to E-couch plus weekly email support, i.e., that these two forms of support will not, effectively, differ in their effectiveness.

• It is also hypothesized that lower initial symptoms, fewer past treatment episodes, fewer intimate relationships, lower education, poorer computer literacy and lower perceived need for treatment will predict increased drop out and reduced adherence.

### Primary Outcome Measure

The primary outcome is the severity of anxiety symptoms, assessed using the GAD-7 scale [43].

### Secondary Outcome Measures

Secondary outcomes include: GAD caseness status at six months post-intervention, as measured by a second administration of the MINI; worry, measured by the Penn State Worry Questionnaire [45]; anxiety sensitivity, as measured by ASI [46]; depression symptoms assessed by the CES-D [47] and PHQ Depression [48]; harmful/hazardous alcohol use as measured by AUDIT [49]; disability, measured by the 'Days Out of Role' questions from the US National Comorbidity Survey and number of hours worked per day [50]; health knowledge using formats previously developed for depression and adapted for anxiety; psychological distress using the K10 [51]; help seeking using scales measuring actions taken to overcome anxiety adapted from parallel depression versions of these [52]. In addition, changes in perceived need for treatment will be assessed by the following item: "Was there ever a time in the last 12 months when you felt that you might need to see a health professional because of problems with your emotions or nerves?" [53].

The following measures will be included to assess outcome predictors and potential mediators of the effectiveness of the intervention. Personal and perceived stigma toward those with GAD will be assessed by a new scale currently under development - the Generalised Anxiety Stigma Scale, symptoms of social phobia will be assessed using the Social Phobia Inventory [54] and a new social phobia screener that is in development, whilst symptoms of panic will be measured using PHQ Panic [55] and a new panic disorder screener that is in development. Availability of social support will be assessed using MOS Social Support Survey and adherence measured by survey return rates and website usage. Preferences for treatment type and expectations of the trial will be assessed using previously developed formats [27]. Predictors of outcome including smoking, medication use, perceived helpfulness of sources, childhood adversity, physical health and life events will use scales developed for the PATH through Life Study [56].

### Subsidiary Outcome Measures

Subsidiary outcomes will be measured. These will include direct costs of each arm to determine the merit of online treatments, satisfaction using previously used self report scales, and reason for drop out which will be assessed using a modified Ritterband's Adherence Interview [57]. In addition, the demographic data reported in the screening phase will be analysed to compare those who responded to the general population.

### Sample Size and Power Calculations

Most treatment trials of CBT based GAD report an effect of approximately .6 SDs relative to placebo and .8 SDs relative to minimal contact [58]. For prevention in adults, Kenardy and colleagues [13,41] reported an effect size change of approximately .6 relative to control condition for cognitions and depression. However, because the same test was used to both select the sample and measure outcome, there may have been regression to the mean, which may have inflated this effect. For the purposes of the present trial, we assume a correlation of .7 between pre- and post-test measurements, and find that the study will have 80% power to detect differences in change from baseline of approximately .3 standard deviations in *a priori *contrasts of trial arms conducted within the framework of an omnibus test of condition by time mixed model repeated measures analysis.

Comparison of email to human support will be undertaken within a non-inferiority/equivalence framework [59]. This will maximize power to detect a statistically significant inferiority of email to human support. For the evaluation of prevention-significant change, there will be 80% power to detect a relative advantage as low as 25% to 60% in the response rate in the prevention compared to placebo depending on baseline response. Greater power may be able to be obtained by including all trial arms (a, b, c) and placebo arms (d, e) in this analysis. Power to detect differences in risk rates for diagnosis of incident GAD is constrained by the large sample required and the time period over which participants will be followed. Nevertheless, incidence rates will be calculated and compared using methods established as being accurate for low rates in moderate sized samples [60]. Other categorical analyses (relative risk reduction, number needed to treat) will be based on the criteria of 20% reduction in symptoms and absence of DSM GAD caseness. This sample size will allow for multivariate analyses with up to six predictors, assuming moderate size effects [61]. In this trial we estimate pre-post effect sizes for Conditions 1-5 to be .5, .15, .8, .2 and .8 SDs, respectively. Differences between active and comparator arms will be detected within this trial with good power, as outlined above. With regard to the examination of factors associated with response, adherence and drop out, allowing for 15% attrition, the study will have 80% power to detect simple associations between variables just below r = 0.3. When predictors are dichotomous, there will be similar power to detect differences just less than 0.6 standard deviations in response between groups.

### Random Allocation Procedure

As required by ICH Guideline E9 [62], randomisation of participants to treatment groups will be carried out under trial biostatisticians who will not be involved in the day to day conduct of the trial. Random allocation to the treatment groups will occur immediately after the baseline interview has been completed. The algorithm for random allocation will consist of a stratified block design, with stratification by level of symptoms, gender, and past diagnosis of GAD and a block size of 10. There will be eight strata (2 × 2 × 2), corresponding to higher/lower symptom level, female/male gender, and previous diagnosis of GAD. Allocation will be administered within the existing software architecture developed by the investigators. Participants will be informed that they have been assigned to a condition after completing the baseline interview, and may begin the first module one week later.

### Statistical Considerations

The senior trial biostatistician will be blinded to the treatment groups being analysed until the analysis has been completed, rendering the statistical analysis masked. Furthermore, no trial biostatisticians will be involved in the allocation of individuals to inventions, administration of treatment, measuring outcomes, entering data, or assessing eligibility of participants.

Primary analyses [43] will be undertaken on an intent-to-treat (ITT) basis, including all participants randomised regardless of treatment actually received or withdrawal from the trial. Mixed-model repeated measures (MMRM) analyses will be used because of the ability of this approach to include participants with missing data without using discredited techniques such as last observation carried forward [63]. For non-inferiority components, appropriate analyses will be undertaken. These will generally not be ITT based, as this model is often anti-conservative in these circumstances [56].

Non-linear mixed models will be used to analyse categorical outcomes including increased caseness status and whether the participant has met the benchmark decrease of 20% from baseline at each of the follow-up assessments on the GAD-7. If necessary, multiple imputation including demographic and other background variables as predictors will be used to allow inclusion of data from all participants and not simply those with data which would permits inclusion in mixed models. Additional analyses will explore participant characteristics which moderate outcome and, if appropriate, levels of presenting severity associated with significant improvement. Other outcomes (such as data on reasons for dropout) will be described.

## Discussion

The WebGAD trial represents an opportunity to test the potential benefit of a population-based preventive intervention for a mental disorder in adults. This will be the first true prevention trial of an indicated GAD prevention intervention in young adults. It will be the first Internet trial for any mental disorder that simultaneously investigates the role of human and automated support and which goes beyond research directed at effectiveness--although this is also a goal--to research focusing on process variables, such as predictors of adherence and of non-response. It will determine direct costs and outcomes with direct relevance to implementation. The large target sample size will permit the development of exploratory predictive models and may enable targeting of modifiable causes of non-response. The E-couch program has been developed on a platform that is immediately scalable, thus making it a practical prevention program. If effective, E-couch could be promoted and disseminated immediately to the population as a whole.

## Status of the Trial

The study will commence in April 2010. To allow sufficient time to implement the intervention, the sample will be recruited in four intake cohorts conducted 2-3 months apart, with the pilot study beginning in June 2010, the second intake cohort beginning in August 2010, in the third intake cohort in October 2010, and the last intake cohort in December 2010. The trial is expected to end in June 2012.

## Competing interests

HC and KMG are directors of e-hub at the ANU which developed the E-couch program. However, neither author derives personal financial benefit from the operation of e-hub.

## Authors' contributions

HC, KMG, AJM, JK, PJB developed the trial protocol and wrote the applications for NHMRC Grant 525419. KK, PJB and KB further developed the details of the trial protocol. KK drafted the manuscript. All authors contributed to the editing of the manuscript and writing of a second draft.

## Author Information

**HC**. Particular expertise in mental health and the use of the Internet in the prevention of mental disorders and has published extensively on and run many trials of Internet interventions.

**KMG**. Extensive research experience in the areas of e-mental health including the development and evaluation of Internet interventions using RCTs. Experienced in overseeing/supervising a public depression ISG (with Ethics approval). Registered psychologist.

**AJM**. Experienced in the quantitative aspects of mental health research. This includes development and analysis of psychometric measures, screening and diagnosis tests, modelling longitudinal data, and the conduct and analysis of controlled trials and interventions in mental health.

**KK**. Trial Manager for the WebGAD prevention trial and Research Assistant to Professor Christensen.

**PJB**. Expertise in statistical analysis and data management of large-scale behavioural research studies, and experience in the design and implementation of longitudinal studies.

**JK**. Professor Kenardy will provide clinical expertise to the project in guiding treatment and assessment procedures and protocol. He has extensive experience in translating clinical treatments into the web medium. He also has specific expertise in the design and execution of clinical trials of psychological interventions.

**CE**. Trial Manager for WebGAD Treatment project at the Brain & Mind Research Institute, University of Sydney.

**KB**. Extensive experience in the design and implementation of online trials of psychological interventions, and the development of online intervention applications including E-couch.

## Pre-publication history

The pre-publication history for this paper can be accessed here:

http://www.biomedcentral.com/1471-244X/10/25/prepub
